# Changes in the Composition of Atlantic Salmon upon the Brown Seaweed (*Saccharina latissima*) Treatment

**DOI:** 10.3390/foods8120625

**Published:** 2019-11-29

**Authors:** Even Moen Kirkholt, Alexander Dikiy, Elena Shumilina

**Affiliations:** Department of Biotechnology and Food Science, Norwegian University of Science and Technology (NTNU), N-7491 Trondheim, Norway; evenmki@stud.ntnu.no (E.M.K.); alex.dikiy@biotech.ntnu.no (A.D.)

**Keywords:** sugar kelp, *Saccharina latissima*, seaweed, salmon, NMR metabolomics

## Abstract

This study shows the potential of improving the taste and shelf life of salmon by storing it in conjunction with sugar kelp. The influence of the addition of wet sugar kelp to Atlantic salmon fillet was assessed using a Nuclear Magnetic Resonance (NMR) metabolomics approach. Seaweed treatment caused significant changes in the polar and non-polar metabolic composition of salmon muscle upon its storage. The mutual diffusion of sugar kelp and salmon metabolites caused a significant decrease of the formation of the off-smelling compound trimethylamine and the biogenic amines, along with an increase of umami-related compounds (aspartate and succinic acid). Carotenoid composition of the seaweed-treated samples significantly differs from the reference samples. The amount of wet seaweeds used for the treatment and the time passed after the fish slaughter influence salmon quality parameters.

## 1. Introduction

Oceans house an enormous amount of marine resources that can be utilized to satisfy the needs of the human population. While the aquaculture industry is well established in the European countries, the production of food products made from seaweeds is mainly limited to additives. For instance, alginate and carrageenan are extracted from seaweeds and used as thickeners in foods [1,2]. In contrast to the Asian countries, where the use of the seaweeds as a food have a long tradition, seaweed dishes are not common in Europe [3]. Therefore, there is a potential for introducing seaweeds and new functional food products containing seaweed to the European cuisine and food industry, which are more sustainable to produce and reduce the strain on land-based agriculture. Seaweeds contain various bioactive compounds, which makes them a source of nutritious biomass that can be utilized for food and feed purposes [4,5,6]. Different methods to obtain biologically active compounds from algal biomass and possible applications of seaweed extracts were described in detail by Michalak and Chojnacka [7]. The number of bioactive compounds isolated from seaweeds has been increasing steadily. Seaweed extracts contain carbohydrates, proteins, minerals, lipids, polyunsaturated fatty acids as well as antioxidants, pigments and other bioactive compounds which have antibacterial, antiviral, antifungal, antioxidative, anti-inflammatory, and antitumor properties [7].

*Saccharina latissima* (*S. latissima*), commonly known as sugar kelp, is a brown seaweed that grows on the Northern Hemisphere. The cultivation of this seaweed species is in constant growth [8]. Generally, brown seaweeds are rich in polysaccharides, minerals, polyunsaturated fatty acids and vitamins [9]. Despite its composition being affected by seasonal changes [10,11], alginate, mannitol, laminarin, cellulose, proteins and salts were reported as the main components of sugar kelp [12]. In addition, *S. latissima* contains a large number of small organic molecules called metabolites. The metabolic profile of *S. latissima* was recently elucidated using liquid chromatography–mass spectrometry [13]. The authors classified all metabolites into seven groups and found that brown algae are second in “amino acids and derivatives” and “carbohydrates” content [13]. Brown algae also had the highest number of “cofactors, prosthetic groups and electron carriers” [13].

The knowledge of the metabolic composition enables targeted use of seaweeds for medical, nutritional or other purposes. Brown algae exhibit anti-oxidant, anti-viral, anti-diabetic and anti-microbial properties [9,14,15,16]. For that reason they can be used as functional food ingredients [17,18]. It was shown that the methanol extract from brown seaweed, *Himanthalia elongata*, can inhibit the growth of the common food spoilage and pathogenic organisms and, therefore, has a good potential as a natural antimicrobial agent for food preservation [15]. In addition, the inhibitory effects of the algae extract on the microbial proliferation in stored fish were reported [15,19].

Seaweed addition might affect the organoleptic properties of food, besides the inactivation of the bacteria growth. Biochemical processes within complex system composed by fish and seaweed may differ from the reference individual products. These changes will reflect on the quality, flavor and shelf life of the seaweed-treated food. Therefore, careful assessment of these parameters should be done in order to ensure palatability and safety of the new complex food.

A Nuclear Magnetic Resonance (NMR) metabolomics approach can be used as an analytical method to describe the metabolic composition and post-mortem changes in Atlantic salmon on a molecular level [20,21,22,23]. Previously, we applied NMR spectroscopy to describe the post-mortem processes that occur within two weeks of storage in Atlantic salmon muscles at 4 °C. More than 30 compounds were detected and quantified using NMR spectroscopy [20].

In this study, the NMR metabolomics were used to define how the addition of *Saccharina lattisima* to salmon will influence the fillet quality and its shelf life.

## 2. Materials and Methods

### 2.1. Experimental Design

Post-mortem changes and shelf life of Atlantic salmon (*Salmo salar*) muscles upon the addition of wet sugar kelp (*Saccharina latissima*) were studied by analyzing samples stored at 4 °C. Reference and treated salmon were stored for three weeks and sampled at different time points. Polar and non-polar metabolites were extracted from salmon by using trichloroacetic acid (TCA) or acetone, respectively. NMR spectroscopy was used for metabolic profiling of the salmon samples. A principal component analysis (PCA) was used to reveal significant differences between reference and seaweed-treated salmon.

It was assessed how changes in the volume of seaweed liquid and the start time of seaweed treatment altered the salmon composition. For that, two experiments were carried out. During the first experiment, seaweed was wrapped around salmon muscle on day 5 after slaughter with a varying volume of dripped liquid. In the second experiment, the seaweed liquid was gently removed, and the salmon was treated with seaweed on day 7 after slaughter.

### 2.2. Chemicals

Deuterium oxide (D_2_O, 99.9%) from Cambridge Isotope Laboratories Inc. (Andover, MA, USA); Trichloroacetic acid (TCA) from Sigma-Aldrich (St. Louis, MO, USA); 3-(Trimethylsilyl)-propionic-2,2,3,3-d4 acid sodium salt (TSP, 98 atom % D) from Armar Chemicals (Dottingen, Switzerland); Acetone from VWR International S.A.S (Fontenay-sous-Bois, France); Acetone-d6 (99 + atom % D) with 0.03% of tetramethylsilane (TMS) from Acros Organics (Morris Plains, NJ, USA); Sodium hydroxide from Sigma-Aldrich was used to adjust the pH of the samples.

### 2.3. Saccharina Latissima Samples

Vacuum-packed and frozen *S. latissima* (further, seaweed) were obtained from Seaweed Energy Solutions AS (SES). The seaweed seedlings had been produced in SES’ hatchery in Trondheim and further brought to SES’ sea farm at Frøya for cultivation and harvesting. Seedlings were deployed in January–February and harvested during the time period of April–May. The seaweeds were stored in flow-through seawater between harvesting and packaging. The buffering period was less than 3 days. The seaweed was rinsed in sterilized seawater before packaging and further frozen at −20 °C on the same day as packaging.

### 2.4. Salmon Sampling

#### 2.4.1. Experiment 1: Salmon storage with seaweed and dripped liquid 

Three packages of SALMA^®^ back loin fillets were purchased at a local grocery store on day 5 after slaughter. Each package was sampled and extracted at the day of purchase to be used as a reference point. Further, each fillet was divided into 14 pieces of about 5 g. Seven of them were wrapped in variable amounts of seaweed containing dripped liquid (further, seaweed-treated samples, *STS*). The ratio of fish muscle to seaweed and dripped liquid in grams was ~5:2.5:2.5, respectively. The remaining seven samples were left in their original state as a reference (further, reference samples, *RS*). The liquid that dripped from the thawed seaweed was collected and stored at −40 °C (further, *juice*). Both STS and RS were stored in zip lock plastic bags at 4 °C for up to three weeks after slaughter. The TCA extraction of polar metabolites from the muscle samples was carried out at days 5, 7, 9, 11, 14, 17, 19 and 21 after slaughter (further, T5, T7, T9, T11, T14, T17, T19 and T21).

#### 2.4.2. Experiment 2: Salmon storage with seaweed and limited amount of dripped liquid

To further evaluate the obtained data, 1.9 kg of Atlantic salmon fillet was purchased from Ravnkloa (Trondheim, Norway) on day 7 after slaughter. Two pieces of about 5 g of salmon back loin were processed immediately. Their polar (TCA extract) and non-polar (acetone extract) compositions at day 7 after slaughter were used as references. Thereafter, seaweed-treated and reference muscle samples for the second experiment (STS-2 and RS-2, respectively) were prepared as in Experiment 1, but the seaweed liquid was gently removed from the algal tissue before the treatment. Twelve samples were prepared during this experiment. The extraction of the polar metabolites by TCA was carried out at days 7, 9, 11, 14, 17, 19 and 21 after slaughter (further, T7, T9, T11, T14, T17 and T21). In addition, another three RS-2 and three STS-2 samples were prepared for the extraction of non-polar metabolites by acetone. Acetone extraction was carried out at days 7, 11, 17 and 21 after slaughter.

### 2.5. Extraction

#### 2.5.1. Extraction of Polar Metabolites from Salmon Muscle by TCA

The variability of metabolites in their chemical activity, polarity and stability necessitates a careful choice of an extraction protocol. A detailed review of the extraction methods used in metabolomics research was performed by Fan [24]. In our study, polar metabolites were extracted with trichloroacetic acid. This extraction method was also used in other studies of salmon composition and is suitable for NMR metabolic analysis [20,22,24]. Polar metabolites were extracted by TCA following a modified protocol [22]. The seaweed that was used for the wrapping (*algal tissue*) and remaining dripped liquid (*algal juice*) were collected from the STS samples and stored at −40 °C. Five grams of salmon muscles were cut into small pieces and homogenized using a Mixer Mill MM 400 (Retsch, Haan, Germany) as follows: 18 stainless 3 mm balls; 5 min; 30 s^−1^ oscillations. Thereafter, 30 mL of ice-cooled 7.5% TCA were added to the homogenate and homogenization was repeated for 4 min with 20 s^−1^ oscillations. The homogenate was centrifuged (10 min; 6800 *g*; 8 °C). The supernatant was separated and filtrated through filter paper (Whatman, Maidstone, UK). The pH of the filtrate was adjusted to pH = 7.0 using 9 M NaOH.

#### 2.5.2. Extraction of Polar Metabolites from *Saccharina Latissima* by Water

A total of 2.8 g of thawed seaweed was vigorously cut with a carbon steel surgical blade and transferred to a 50 mL centrifuge tube. A quantity of 15 mL of deionized water was added to the sample, followed by mixing of the sample for 3 h using a nutation mixer at room temperature. The sample was centrifuged (10 min; 6800 *g*), and the resulting supernatant was separated and filtrated through filter paper. The pH of the filtrate was adjusted to pH = 7.0 using 9 M NaOH.

#### 2.5.3. Extraction of Non-Polar Metabolites from Salmon Muscle by Acetone

A total of 5 g of salmon muscle was cut into small pieces and homogenized using a Mixer Mill MM 400 (Retsch, Haan, Germany) as follows: 18 stainless 3 mm balls; 5 min; 30 s^−1^ oscillations. Thereafter, 15 mL of ice-cooled acetone were added to the homogenate and the homogenization was repeated for 4 min with 20 s^−1^ oscillations. The homogenate was centrifuged (10 min; 6800 *g*; 8 °C). The supernatant was decanted and collected. Acetone and trace amounts of water were removed from the sample by consecutive use of the SpeedVac Vacuum Concentrator (Thermo Fischer Scientific, Waltham, MA, USA) and Alpha 1–4 LO plus laboratory freeze-dryer (CHRIST, Osterode am Harz, Germany). The dried samples were stored at −40 °C before analysis.

### 2.6. NMR Sample Preparation

#### 2.6.1. Salmon Muscle and Algal Polar Extracts

A quantity of 540 µL of extract was mixed with 60 µL of 1 mM TSP in 20 mM sodium phosphate buffer, pH 7 in D_2_O. The samples were centrifuged for 5 min at 20 °C and 20,000 g. A quantity of 530 µL of the supernatant was transferred to a standard 5 mm NMR tube.

#### 2.6.2. Algal Juice

The collected algal juice was centrifuged at 20,000 *g* for 5 min at 20 °C. A quantity of 540 µL of the resulting supernatant was mixed with 60 µL of 1 mM TSP in 20 mM sodium phosphate buffer, pH = 7.0 in D_2_O. The volume of the resulting solution was adjusted to 540 µL by adding deionized water. A quantity of 530 µL of the mixed solution was transferred to a standard 5 mm NMR tube.

#### 2.6.3. Salmon Muscle Acetone Extract

Dry samples were solubilized in 600 µL of acetone-d6. The solution was centrifuged for 5 min at 20,000 g. The resulting supernatant was transferred to a standard 3 mm NMR tube.

### 2.7. NMR Data Acquisition and Processing

1D ^1^H NMR spectra of all extracts were acquired at 300 K with a Bruker Avance 600 MHz spectrometer equipped with a 5 mm z-gradient TXI (H/C/N) cryoprobe. The NMR spectra of the polar extracts were acquired using the Bruker pulse sequences *noesygppr1d* with the following acquisition parameters: NS = 48; SW = 20 ppm; O1 = 2820 Hz; D1 = 4 s. RG = 144 and 50.8 were used for the TCA and seaweed samples, respectively. The NMR spectra of the acetone extracts were acquired with the Bruker pulse sequences *zg30* and the following acquisition parameters: NS = 512; SW = 20 ppm; RG = 2.8; O1 = 2820 Hz; D1 = 4 s. The NMR spectra were processed with TopSpin 3.6.1 (Bruker, Rheinstetten, Germany). The metabolite assignment was performed by using our previous studies [20,22] and published reference standards (*Biological Magnetic Resonance Data Bank* and *Human Metabolome Database*) [25,26]. The spectra of TCA and acetone extracts were calibrated to the resonance of either the TSP or TMS signal, respectively. In addition, the phase and baseline in the spectra of acetone extracts were corrected manually.

The quantification of the polar metabolites was carried out using the reference integral of the TSP signal and integrals of the metabolites’ resonance signals. The value used for the quantification was an average integral out of three resonance integrations.

### 2.8. Statistical Data Analysis

A Principal Component Analysis (PCA) of the spectroscopic data was performed to determine the differences in the metabolic profiles between reference and seaweed-treated samples as follows. First, all the spectra were normalized to an average mass of extracted tissue and volume of extractions. The spectra of the TCA extracts were manually binned into non-regular buckets in Amix-Viewer, version 4.0 (Bruker, Rheinstetten, Germany). The table containing the integral values of the defined buckets for all samples, was further transported to IBM SPSS Statistics version 25 (Armonk, New York, USA) for an ANOVA analysis (*p*-value < 0.05). This analysis allowed to define the buckets or spectral regions whose values significantly differed between the RS and STS samples. The buckets for which the p-values exceeded 0.05 were removed from the following PCA analysis. The reduced table was further imported to Unscrambler X version 10.5 (CAMO Software AS, Oslo, Norway), where the PCA analysis was performed.

## 3. Results and Discussion

### 3.1. Principal Component Analysis

This study assessed the metabolic response of salmon muscle to the addition of seaweed during storage (Experiment 1). For this purpose, the recorded ^1^H NMR spectra were binned into 96 buckets. The initial number of buckets was reduced to 45 significant buckets by the ANOVA test (see Statistical data analysis in Materials and Methods). The *p* and *f* values for the 45 significant variables are shown in Table 1.

**Figure 1:**
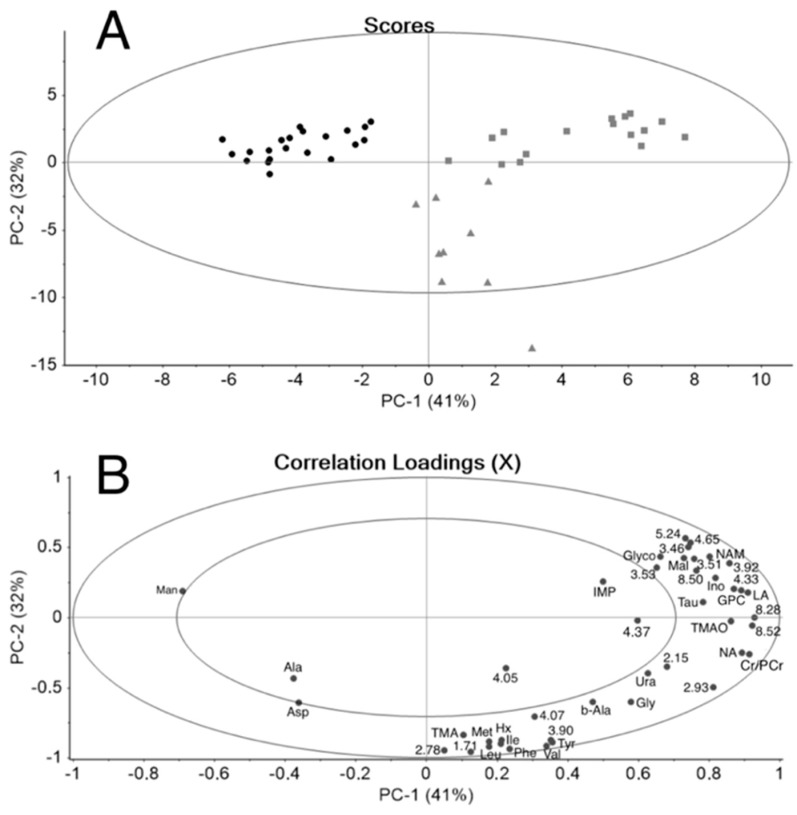
Score plot (**A**) and correlation loadings (**B**) of mean-centered PCA performed using the reduced bucket table of ^1^H NMR spectra of salmon muscle TCA extracts. Score plot: black circles—STS samples; gray squares—RS stored between T5–T14; gray triangles—RS stored until T17–T21. Correlation loadings marked with numbers in B represent chemical shifts of unknown compounds in ppm. Abbreviations used in figure: Ala—alanine; Asp—aspartic acid; b-Ala—β-Alanine; Cr/PCr—creatine/phosphocreatine; GPC—glycerophosphocholine; Gly—glycine; Glyco—glycogen; Hx—hypoxanthine; IMP—inosine-5′-monophosphate; Ino—inosine; Ile—isoleucine; LA—lactic acid; Leu—leucine; Mal—maltose; Man—mannitol; Met—methionine; NA—nicotinic acid; NAM—niacinamide; Phe—phenylalanine; Tau—taurine; Thr—threonine; TMA—trimethylamine; TMAO—trimethylamine oxide; Tyr—tyrosine; Ura—uracil; Val—valine.

The integrals of the significant buckets were subsequently used in the PCA. Figure 1 shows the resulting PCA scores and correlation loadings plots.

A distinctive horizontal separation of RS and STS clusters can be observed in the scores plot A. Clustering of the samples indicates the similarity of the post-mortem processes within each group. At the same time, the distancing of the two groups indicates significant differences in the metabolomes caused by the addition of the seaweed. A vertical spread of the clusters is more obvious in the RS samples and is related to the metabolic changes during the storage. The larger contribution of storage time on the samples’ distribution in the RS group indicates that some post-mortem processes are inhibited in the STS samples. The correlation loadings plot (Figure 1B) shows good clustering of the buckets/variables. The variables on the left side of the correlation loadings plot indicate the metabolites that are present at significantly higher concentrations in the STS samples. Aspartic acid, alanine and mannitol are observed to be the main ones. The right side of the correlation loadings plot contains the metabolites whose concentrations were significantly higher in the RS samples: IMP, glycogen, maltose, inosine, lactic acid, TMAO and creatine. Storage causes several changes in the metabolic composition of the reference samples and this is reflected in the vertical distribution of the RS samples stored until day 17–21 after slaughter (Figure 1A, gray triangles). The metabolites on the right-bottom side of the loading plot (Figure 1B) were found at elevated concentrations in the RS samples stored until day 17–21 after slaughter. Such changes are the result of the post-mortem processes due to the autolysis and bacteriological activity that take place in the stored reference salmon muscle and were described in detail earlier [20,21,27]. Briefly, the concentrations of anserine, phospho/creatine and niacinamide in the reference samples do not change significantly during storage [20]. The behavior of IMP, inosine and hypoxanthine upon storage reflects the ATP degradation. This process has been found to correlate with the loss of fish freshness [28]. The formation of TMA and the increase in the concentration of the amino acids during the prolonged storage (day 17–21) occur due to the increased bacteriological activity.

In conclusion, the post-mortem metabolic changes in reference salmon muscle are in good accordance with the previous findings [20,21]. In contrast, seaweed-treated salmon samples are placed on the opposite part of the scores plot and do not have a vertical distribution. This is a sign that the seaweed treatment influences the post-mortem processes in salmon during storage.

### 3.2. 3-Part System

Seaweed-treated salmon muscles represent a single system consisting of three parts: fish muscle, algal tissue and dripped algal liquid (juice). The metabolites belonging to different parts reach an equilibrium concentration, which is due to mutual diffusion through the connective phase (juice) during storage. Such diffusion causes a decrease in the concentration of the fish’s own metabolites, simultaneously with the enrichment of compounds from the seaweed. The same applies to the other components of the system, i.e., algal juice and tissue. To monitor metabolic changes in the three-part system, the reference compositions of each part should be considered. The metabolic composition of Atlantic salmon and its changes upon storage have previously been described. In this chapter, we will describe the polar metabolic composition of the seaweed tissue and dripped liquid.

#### 3.2.1. Polar Metabolites from Sugar Kelp and Its Juice

**Figure 2:**
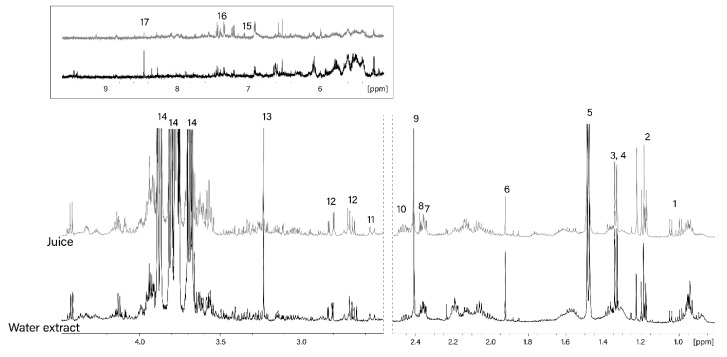
1D ^1^H NMR spectra of *Saccharina latissima* juice (gray) and water extract (black). 1—valine, isoleucine, leucine; 2—ethanol; 3—lactate; 4—threonine; 5—alanine; 6—acetate; 7—glutamate; 8—pyruvate; 9—succinate; 10—glutamine; 11—citrate; 12—aspartate; 13—glycerophosphocholine; 14—mannitol; 15—tyrosine; 16—phenylalanine; 17—formate.

The NMR spectrum of the water extract of sugar kelp is shown in Figure 2.

The main polar metabolites extracted from the algae were mannitol, glutamate, alanine, glycerophosphocholine (GPC), aspartate, valine and other metabolites, which are listed in Figure 2. The concentration of the main metabolites is shown in Table 2.

The obtained data is in good agreement with the previous findings of Belghit et al. [13]. The authors also listed mannitol, glutamate, alanine and GPC as the most abundant metabolites of sugar kelp. Despite minor differences (mostly in the aromatic region from 5–9 ppm), the seaweed juice has a rather similar polar metabolic composition to the seaweed extract (Figure 2).

#### 3.2.2. Changes in the Salmon Metabolome after the Addition of Sugar Kelp

Storing salmon muscles wrapped in seaweed for 48 h leads to an equilibrium distribution of polar metabolites between all parts of the three-part system. Thirty compounds were assigned and quantified in the salmon extract to compare the metabolites changes in reference and seaweed-treated samples (Table 3).

Comparing the STS and RS composition on T7 (48 h after the treatment) showed a significant decrease of characteristic fish metabolites such as phospho/creatine (further, creatine), anserine, trimethylamine oxide (TMAO), lactate or products of ATP degradation. The concentration of creatine in STS samples decreased at least two-fold compared to RS (for example, from 3.99 to 1.48 mmoL/100 g, Table 3). The concentration of creatine in STS samples remained stable after 48 h of seaweed-treatment. The same trends were observed for anserine and TMAO. The concentration of anserine and TMAO in STS samples on T7 was decreased at least two-fold compared to RS samples, and they remained stable after 48 hours of seaweed-treatment. These results indicate that the system of salmon muscle, seaweed tissue and juice had reached an equilibrium in metabolite distribution at the first time point of sampling in the experiment (48 h after the addition of seaweed).

The NMR spectra of STS also contained resonance signals of mannitol, aspartate and other molecules diffused from the sugar kelp. The concentration of mannitol varied from 112.7 to 642.2 mg/100 g fish between parallels of STS samples and between the time of sampling. It is suggested that the concentration differences were heavily influenced by the amount of applied seaweed tissue and juice on the salmon in Experiment 1.

The decrease in the salmon metabolites is not as dramatic in Experiment 2, where the volume of seaweed juice was reduced. In Experiment 1, the concentration of creatine in STS samples at T7 was decreased at least two-fold compared to RS samples, while in Experiment 2, the concentration at T9 in STS-2 was only slightly reduced compared to RS-2 (from 3.64 to 3.27 mmoL/100 g of fish, Table 3). This can be explained by the reduced amount of seaweed juice that was applied during the second experiment. Therefore, the amount of seaweed juice used during the salmon treatment might significantly change the taste of the final product.

The influence of the metabolite diffusion on the fillets flavor and shelf life will be discussed further in Section 3.3 and Section 3.4.

### 3.3. Shelf Life of Atlantic Salmon

#### 3.3.1. Formation of Trimethylamine (TMA)

The formation of TMA in fish gives rise to an unpleasant odor and it is, therefore, related to fish quality [29]. Salmon treatment with seaweed significantly reduced the formation of this compound. The amount of up to 4.2 mg TMA/100 g fish is used to describe prime quality fish [30]. The RS samples were below this limit up to T14, while the STS samples did not exceed this level for the whole storage period up to T21. STS contained significantly less TMA compared to RS samples. The TMA concentration in STS (parallel 2) was reduced by 3.9 times compared to RS at T7 (48 h after the treatment). The TMA concentration in STS samples were reduced by at least 90% compared to RS at T21.

To explain these findings, the concentration of TMAO was assessed in RS and STS samples. TMAO might be reduced by bacteria to form TMA. The concentration of TMAO in RS samples was relatively stable up to T14, and it decreased after this time. This corresponded to an increase in the TMA levels within the samples. In contrast, the amount of TMAO in STS samples, after a sharp decrease due to diffusion on T7, remained relatively stable during the whole storage period.

In Experiment 2, the concentration of TMAO was similar in the RS-2 and STS-2 samples and continuously decreased during storage. This might be caused by two factors. Firstly, due to the reduced diffusion, TMAO was not removed from the STS-2 samples. Secondly, the addition of seaweed was performed at a later stage of storage with an already advanced stage of bacterial spoilage. Therefore, the inhibition of TMAO reduction was not as efficient as in Experiment 1. However, the effect of seaweed addition on the formation of TMA was still pronounced. During early storage, the amount of TMA in RS-2 and STS-2 samples was rather similar. Its concentration was about 1.5 times higher in RS-2 on T21. Therefore, the addition of seaweed to salmon does not only reduce the amount of TMAO in the muscle due to diffusion, but it also inhibits the bacterial reduction of the remaining TMAO to TMA.

#### 3.3.2. Formation of Biogenic Amines

Biogenic amines are potentially toxic, if their concentration in foods exceed given threshold levels [31]. Biogenic amines, such as tyramine, putrescine and cadaverine are usually formed by microbial decarboxylation of their amino acid precursors [31]. Paulsen et al. suggested 95 mg/100 g as a maximum tolerable level of tyramine in fish [32]. The concentration of tyramine in both experiments did not exceed 5 mg/100 g of fish, which is well below the mentioned maximum tolerable level. The formation of tyramine during Experiment 1 was only detected in parallel 3 of the RS sample at T21. The formation of tyramine in Experiment 2 was observed between T11–T21 in both RS-2 and STS-2 samples.

Rauscher-Gabernig et al. suggested the maximum tolerable level of putrescine to be set to 17 mg/100 g fish [33]. No formation of putrescine was observed in the STS samples, while it was detected in RS samples between T17–T21 during Experiment 1. Due to overlapping of the putrescine resonance signals with cadaverine, the former biogenic amine was not quantified in the first experiment. However, putrescine was qualitatively observed in the reference spectra. Nevertheless, it was possible to quantify putrescine in Experiment 2, where its concentration did not exceed 9 mg/100 g. The formation of putrescine in STS-2 was reduced compared to the RS-2. The formation of cadaverine was detected only in RS from T17 during Experiment 1. During Experiment 2, cadaverine was observed in both RS-2 and STS-2 samples from T11. The difference in the formation of biogenic amines might be a result of the different stages of bacterial spoilage of the salmon fillets in the two experiments.

### 3.4. Flavor Enrichment

The treatment of the salmon fillets with wet algae caused not only a decrease in salmon metabolites, but at the same time, enriched the muscle with compounds that were present in the algae (as was discussed in Section 3.2). Some of the molecules that can enhance the original flavor of salmon will be discussed in this chapter.

Aspartate is considered as an umami-related substance that can enhance food flavor [34]. Within the commercial shelf life of salmon (14 days), aspartate was detected only in STS samples. Its concentration in samples depended on the amount of seaweed used during the treatment. In the case of using large amounts of wet seaweed, the content of aspartate reached 6.06 mg/100 g of salmon in Experiment 1.

Succinate is present in the sugar kelp or can be a potential product of mixed acid fermentation of sugars by bacteria [35]. It is also considered as an umami-related compound [34]. The amount of succinate was increased by at least 22 times in STS compared to RS samples at T7. Succinate was detected in all STS samples between T7–T14. Its concentration in RS increased significantly from day 17–21 and reached up to 21.26 mg/100 g in RS samples at T21. Such an increase might be due to the bacterial spoilage and fermentation processes. The results indicate that the addition of seaweed increases the concentration of succinate in fish due to the metabolite diffusion. In Experiment 2, the concentration differences of succinate between RS-2 and STS-2 samples were smaller. The more advanced stage of bacterial spoilage and the reduced amount of applied juice in Experiment 2 might explain the similar trend of succinate observed in both STS-2 and RS-2.

Glutamate (Glu) is the main contributor to the umami taste [34]. The variations of glutamate concentration during salmon storage have two different trends: variation before and after commercial shelf life (up to 14 days after slaughter). The concentrations of Glu were generally lower in STS compared to RS samples during storage until T11 due to its diffusion into seaweed juice and tissue. The concentrations of Glu in Experiment 2 showed only a slight decrease in STS-2 compared to RS-2 between T7–T11.

The degradation of adenosine triphosphate (ATP) and its catabolites are autolytical changes that occur in fish post-mortem. ATP catabolites include adenosine diphosphate (ADP), adenosine monophosphate (AMP), inosine-5′-monophosphate (IMP), inosine, hypoxanthine, xanthine and uric acid [36]. IMP gives the fresh fish flavor that is found in high-quality seafood [36], while inosine and hypoxanthine are described to give bitter taste and off-flavor of spoiled fish, respectively [37,38].

As discussed in Section 3.2, the addition of seaweed during storage causes diffusion of the metabolites that reduces the amount of all salmon metabolites. Therefore, the amounts of ATP catabolites in salmon muscle will also be reduced after the addition of seaweed. The decrease of ATP or IMP, which depends on the day after slaughter the seaweed is applied, will further cause decrease of catabolites of more advanced degradation, such as hypoxanthine. The concentration of hypoxanthine in STS was significantly reduced compared to RS samples between T7–T21. The hypoxanthine concentration was reduced by at least 2.5 times compared to RS samples in STS samples at T7, and the concentration remained relatively stable for the whole storage period. A reduced concentration of hypoxanthine in salmon could result in improved taste, as bitter off-flavors from hypoxanthine will be reduced. In Experiment 2, the concentration of hypoxanthine in STS-2 samples was not reduced compared to RS-2 until T11. The concentration of hypoxanthine in STS-2 was reduced 1.9 times compared to RS-2, and the concentration remained reduced and relatively stable compared to RS-2 samples between T11–T21.

### 3.5. Carotenoids

**Figure 3:**
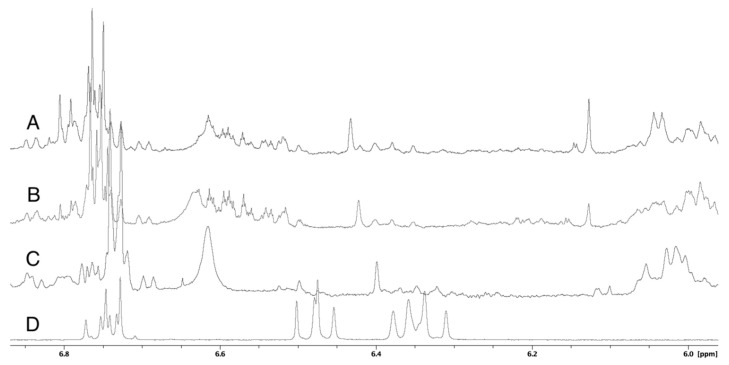
Comparison of ^1^H NMR spectra of salmon acetone extracts. (**A**)—STS-2 at T17; (**B**)—STS-2 at T21; (**C**)—RS-2 at T21; (**D**)—astaxanthin reference.

The carotenoid astaxanthin defines the pinkish-orange appearance of salmon muscle tissue [39]. An increasing difference in the pigmentation of STS and RS samples was observed with storage time. The seaweed-treated samples were pale yellow at the end of the storage time, while the reference samples kept their original color. The difference in pigmentation was more pronounced in the first experiment, when more seaweed was used for the wrapping. The non-polar compounds including carotenoids were extracted with acetone from the STS-2 samples. Figure 3 shows the carotenoid region between 6.0–6.8 ppm of the ^1^H NMR spectrum of STS-2 and RS-2 samples at 17–21 days of storage and reference spectrum of astaxanthin. This region contains the resonance signals of the carotenoids polyenic chain. A significant difference between the seaweed-treated and reference samples is visible between 6.5–6.7 ppm. Therefore, the seaweed treatment causes a change in the carotenoid composition of salmon that is also reflected on the pigmentation of the treated salmon fillet.

## 4. Conclusions

The influence of the addition of wet sugar kelp to Atlantic salmon fillet regarding fish flavor and safety was assessed using an NMR metabolomics approach. Seaweed treatment caused significant changes in the polar and non-polar metabolic composition of salmon muscle upon its storage. Mutual diffusion reduces the concentration of its own metabolites and enriches the salmon muscles with algal components. This leads to the removal of the TMA precursor, TMAO, from the fillet of salmon, and consequently, prevents the formation of an unpleasant odor during storage. Our studies show that the addition of seaweed to salmon inhibits the formation of biogenic amines during fish storage and, therefore, improves the quality of salmon. In addition, seaweed-treated samples are enriched with aspartate, an amino acid that enhances the umami flavor of the fish. The rate of the diffusion and, therefore, decrease or increase of certain metabolites within the salmon muscle depends on the amount of seaweed and juice that are used for the wrapping.

The addition of seaweed might inhibit bacterial growth if applied during an early stage of storage. Prolonged storage of salmon wrapped in wet algae causes changes in the salmon pigmentation and results in pale fillets. This fact should be taken into consideration when *S. lattissima* is used to preserve or enhance the flavor of salmon flesh. Further studies on the use of different seaweeds could provide insight into what species is most favorable to enhance the taste or improve the shelf life of salmon during storage.

## Figures and Tables

**Table 1:** Summary of significant buckets (*p* < 0.05), relative signal assignments, significance levels and F values from ANOVA analysis. Abbreviations: ppm—parts per million; RS—reference salmon; STS—sugar kelp-treated salmon; TMA—trimethylamine; TMAO—trimethylamine oxide; IMP—Inosine-5′-monophosphate.

Chemical Shift of Bucket (ppm)	Compound Assignment	*p* Value	*f* Value
**Cluster of RS**			
0.96	Leucine	0.010	7.188
1.01	Isoleucine	0.009	7.488
1.04	Valine	0.000	19.307
1.71	−	0.009	7.457
2.14	Methionine	0.028	5.138
2.15	−	0.000	23.691
2.56	β-Alanine	0.000	38.144
2.78	−	0.031	4.937
2.90	TMA	0.007	8.018
2.93	−	0.000	416.485
3.04	Phospho/creatine	0.000	1046.988
3.23	Glycerophosphocholine	0.000	40.301
3.27	TMAO	0.000	115.006
3.42	Taurine	0.000	23.640
3.46	−	0.011	6.957
3.51	−	0.005	8.856
3.53	−	0.018	6.081
3.56	Glycine	0.000	35.586
3.90	−	0.000	27.242
3.92	−	0.000	25.875
4.05	−	0.002	11.365
4.07	−	0.000	30.336
4.12	Lactic acid	0.000	93.297
4.28	Inosine	0.000	33.667
4.33	−	0.000	30.980
4.37	−	0.001	13.929
4.65	−	0.009	7.439
5.24	−	0.018	6.013
5.38	Glycogen	0.020	5.792
5.40	Maltose	0.005	8.540
7.19	Tyrosine	0.000	17.817
7.43	Phenylalanine	0.001	11.838
7.55	Uracil	0.000	17.794
7.60	Niacinamide	0.000	29.455
8.20	Hypoxanthine	0.000	16.858
8.28	−	0.000	103.470
8.50	−	0.000	19.896
8.52	−	0.000	136.123
8.58	IMP	0.017	6.122
8.94	Nicotinic acid	0.000	734.601
**Cluster of STS**			
1.48	Alanine	0.013	6.731
2.80	Aspartic acid	0.038	4.578
3.80	Mannitol	0.000	73.052

**Table 2:** Polar composition of *Saccharina latissima* water extract.

Compound	Concentration (mg/100 g)
Acetate	1.62 ± 0.00
Alanine	52.78 ± 0.01
Aspartate	5.70 ± 0.00
Citrate	4.31 ± 0.00
Ethanol	2.57 ± 0.00
Formate	0.30 ± 0.00
Glutamate	13.57 ± 0.01
Glutamine	4.66 ± 0.00
Glycerophosphocholine (GPC)	6.37 ± 0.00
Isoleucine	0.60 ± 0.00
Mannitol	644.92 ± 0.02
Phenylalanine	0.79 ± 0.00
Pyruvate	0.03 ± 0.00
Succinate	7.01 ± 0.00
Tyrosine	0.55 ± 0.00
Valine	1.55 ± 0.00

**Table 3:** Composition of the Atlantic salmon muscle stored at 4 °C. Abbreviations: RS—reference salmon; STS—sugar kelp-treated salmon; Par.—parallels; *-The concentration could not be quantified due to a low signal-to-noise ratio (S/N < 10); **-The concentration could not be quantified due to an overlap of resonance signals.

Concentration (mg/100 g)																
Days after Slaughter																
**Experiment 1**																
**Compound**	**Par.**	**5**	**7**		**9**		**11**		**14**		**17**		**19**		**21**	
		**RS**	**RS**	**STS**	**RS**	**STS**	**RS**	**STS**	**RS**	**STS**	**RS**	**STS**	**RS**	**STS**	**RS**	**STS**
Alanine	1	35.82 ± 0.01	39.76 ± 0.01	74.68 ± 0.01	40.45 ± 0.01	48.85 ± 0.00	45.89 ± 0.00	49.65 ± 0.00	35.12 ± 0.01	75.45 ± 0.01	39.96 ± 0.01	62.81 ± 0.01	32.73 ± 0.01	31.62 ± 0.00	58.30 ± 0.03	42.69 ± 0.01
	2	28.70 ± 0.01	32.22 ± 0.01	48.95 ± 0.01	32.82 ± 0.01	43.70 ± 0.01	39.71 ± 0.00	50.02 ± 0.01	31.67 ± 0.00	58.00 ± 0.01	39.31 ± 0.01	57.76 ± 0.00	46.83 ± 0.03	50.77 ± 0.01	55.80 ± 0.01	55.74 ± 0.01
	3	38.78 ± 0.01	42.63 ± 0.02	50.95 ± 0.01	48.96 ± 0.01	81.42 ± 0.02	43.20 ± 0.02	58.45 ± 0.00	43.65 ± 0.00	73.09 ± 0.00	25.99 ± 0.01	61.51 ± 0.01	39.84 ± 0.00	87.92 ± 0.01	62.04 ± 0.00	39.03 ± 0.00
Anserine	1	671.55 ± 0.01	728.22 ± 0.01	304.35 ± 0.02	714.22 ± 0.00	319.10 ± 0.01	700.59 ± 0.03	223.65 ± 0.01	572.00 ± 0.01	292.02 ± 0.00	631.43 ± 0.00	194.52 ± 0.01	580.65 ± 0.01	216.93 ± 0.01	554.70 ± 0.02	244.89 ± 0.01
	2	652.74 ± 0.00	565.03 ± 0.02	252.94 ± 0.01	575.58 ± 0.00	305.21 ± 0.00	617.29 ± 0.00	218.53 ± 0.01	539.32 ± 0.01	263.32 ± 0.01	570.32 ± 0.01	220.17 ± 0.01	593.39 ± 0.01	127.99 ± 0.01	520.58 ± 0.00	200.97 ± 0.00
	3	774.76 ± 0.03	751.42 ± 0.01	341.72 ± 0.01	757.25 ± 0.01	347.63 ± 0.01	687.16 ± 0.01	205.95 ± 0.02	647.99 ± 0.01	198.59 ± 0.01	637.31 ± 0.01	194.94 ± 0.03	574.51 ± 0.01	208.04 ± 0.00	600.39 ± 0.00	195.33 ± 0.01
Aspartate	1	0.00	0.00	4.17 ± 0.00	0.00	3.30 ± 0.00	0.00	1.67 ± 0.00	0.00	3.79 ± 0.00	0.00	3.19 ± 0.00	1.61 ± 0.00	0.00	0.00	0.00
	2	0.00	0.00	3.37 ± 0.00	0.00	2.78 ± 0.00	0.00	3.34 ± 0.000	0.00	5.47 ± 0.000	0.00	3.54 ± 0.00	0.00	0.96 ± 0.00	3.20 ± 0.00	0.00
	3	0.00	0.00	2.60 ± 0.01	0.00	2.14 ± 0.00	0.00	3.64 ± 0.00	0.00	6.06 ± 0.00	0.00	0.89 ± 0.00	0.00	1.91 ± 0.00	0.00	0.64 ± 0.01
β-Alanine	1	3.35 ± 0.00	5.00 ± 0.00	4.48 ± 0.00	12.45 ± 0.01	2.87 ± 0.00	19.48 ± 0.00	2.30 ± 0.00	18.97 ± 0.01	3.68 ± 0.00	14.36 ± 0.00	8.99 ± 0.00	14.27 ± 0.00	5.62 ± 0.01	22.51 ± 0.01	4.17 ± 0.00
	2	2.58 ± 0.00	4.77 ± 0.00	1.08 ± 0.00	6.61 ± 0.00	3.37 ± 0.00	9.04 ± 0.00	2.37 ± 0.00	13.17 ± 0.00	4.09 ± 0.00	11.73 ± 0.00	5.16 ± 0.00	8.53 ± 0.00	4.44 ± 0.00	12.97 ± 0.00	4.58 ± 0.00
	3	2.57 ± 0.00	4.95 ± 0.00	1.52 ± 0.00	9.32 ± 0.00	4.31 ± 0.00	11.85 ± 0.00	1.99 ± 0.00	16.77 ± 0.00	3.65 ± 0.00	6.88 ± 0.00	5.89 ± 0.00	9.99 ± 0.00	4.55 ± 0.00	14.46 ± 0.00	4.85 ± 0.00
Betaine	1	8.58 ± 0.00	10.00 ± 0.00	2.41 ± 0.00	10.46 ± 0.00	4.46 ± 0.00	12.52 ± 0.00	3.68 ± 0.00	14.42 ± 0.00	4.10 ± 0.00	22.06 ± 0.00	3.86 ± 0.00	29.49 ± 0.01	7.70 ± 0.00	28.82 ± 0.00	8.92 ± 0.00
	2	7.21 ± 0.00	6.81 ± 0.00	2.31 ± 0.00	7.18 ± 0.00	3.62 ± 0.00	7.14 ± 0.00	2.41 ± 0.00	12.82 ± 0.00	4.01 ± 0.00	27.85 ± 0.00	3.40 ± 0.00	36.83 ± 0.01	2.59 ± 0.00	47.02 ± 0.00	9.89 ± 0.00
	3	8.02 ± 0.01	10.06 ± 0.00	3.42 ± 0.00	10.26 ± 0.00	5.29 ± 0.00	9.84 ± 0.01	2.53 ± 0.00	10.16 ± 0.00	2.75 ± 0.00	38.93 ± 0.00	6.80 ± 0.00	32.35 ± 0.00	4.73 ± 0.00	40.16 ± 0.01	9.16 ± 0.00
Creatine (mmol/100 g)	1	3.77 ± 0.15	3.99 ± 0.02	1.48 ± 0.01	3.96 ± 0.06	1.57 ± 0.02	3.37 ± 0.03	1.07 ± 0.02	3.09 ± 0.05	1.41 ± 0.05	3.58 ± 0.03	1.15 ± 0.01	3.38 ± 0.02	1.32 ± 0.00	3.02 ± 0.09	1.31 ± 0.05
	2	3.63 ± 0.08	3.51 ± 0.01	1.36 ± 0.02	3.41 ± 0.02	1.59 ± 0.02	3.43 ± 0.02	1.09 ± 0.02	3.32 ± 0.03	1.46 ± 0.02	3.43 ± 0.04	1.19 ± 0.06	3.70 ± 0.00	0.74 ± 0.01	3.01 ± 0.00	1.22 ± 0.02
	3	3.47 ± 0.00	3.76 ± 0.03	1.52 ± 0.00	3.97 ± 0.07	1.62 ± 0.04	3.55 ± 0.01	0.96 ± 0.03	3.43 ± 0.04	0.95 ± 0.01	3.18 ± 0.11	1.00 ± 0.01	3.04 ± 0.11	1.03 ± 0.02	2.95 ± 0.10	1.01 ± 0.01
Glutamate	1	15.60 ± 0.01	19.03 ± 0.01	18.74 ± 0.00	19.14 ± 0.00	9.92 ± 0.00	22.76 ± 0.00	9.67 ± 0.00	4.73 ± 0.00	13.49 ± 0.01	1.61 ± 0.00	19.09 ± 0.00	4.61 ± 0.00	2.25 ± 0.00	2.87 ± 0.00	2.48 ± 0.00
	2	13.95 ± 0.00	19.13 ± 0.00	12.31 ± 0.00	15.54 ± 0.01	11.24 ± 0.00	18.13 ± 0.00	13.35 ± 0.00	5.23 ± 0.00	18.78 ± 0.00	1.83 ± 0.00	16.62 ± 0.00	5.16 ± 0.00	3.26 ± 0.00	14.23 ± 0.00	3.17 ± 0.00
	3	21.19 ± 0.00	21.00 ± 0.00	12.09 ± 0.00	21.15 ± 0.00	14.94 ± 0.00	27.49 ± 0.00	13.85 ± 0.00	12.62 ± 0.00	18.22 ± 0.00	4.38 ± 0.01	5.40 ± 0.00	6.07 ± 0.00	9.11 ± 0.00	5.41 ± 0.00	4.01 ± 0.00
Glycero-phospho-choline	1	**	71.79 ± 0.004	25.41 ± 0.005	**	**	**	**	**	**	**	**	**	**	**	**
	2	**	56.07 ± 0.003	28.42 ± 0.001	**	**	**	**	**	**	**	**	**	**	**	**
	3	**	82.68 ± 0.000	34.01 ± 0.000	**	**	**	**	**	**	**	**	**	**	**	**
Glycine	1	12.92 ± 0.00	15.00 ± 0.00	3.88 ± 0.00	14.25 ± 0.00	3.27 ± 0.00	13.46 ± 0.01	3.78 ± 0.01	11.09 ± 0.01	5.47 ± 0.00	11.48 ± 0.00	2.98 ± 0.00	11.14 ± 0.00	3.38 ± 0.00	15.31 ± 0.00	3.97 ± 0.00
	2	11.94 ± 0.00	12.49 ± 0.00	3.80 ± 0.00	11.18 ± 0.00	4.86 ± 0.00	14.36 ± 0.00	4.31 ± 0.00	10.75 ± 0.01	4.72 ± 0.00	11.90 ± 0.00	3.82 ± 0.00	16.00 ± 0.00	1.85 ± 0.00	25.42 ± 0.00	4.23 ± 0.00
	3	14.42 ± 0.00	16.17 ± 0.01	4.79 ± 0.00	16.06 ± 0.02	6.57 ± 0.00	16.40 ± 0.01	2.64 ± 0.00	13.95 ± 0.00	4.62 ± 0.00	13.94 ± 0.00	3.97 ± 0.00	16.31 ± 0.00	5.02 ± 0.00	21.84 ± 0.00	3.62 ± 0.00
Hypoxanthine	1	7.16 ± 0.00	20.00 ± 0.00	6.57 ± 0.00	15.00 ± 0.00	6.14 ± 0.00	20.54 ± 0.00	5.98 ± 0.00	20.82 ± 0.00	6.70 ± 0.00	34.04 ± 0.00	8.26 ± 0.00	49.65 ± 0.00	9.64 ± 0.00	62.02 ± 0.00	7.42 ± 0.00
	2	7.62 ± 0.00	18.52 ± 0.00	7.55 ± 0.00	10.32 ± 0.00	5.73 ± 0.00	13.68 ± 0.00	5.28 ± 0.00	15.00 ± 0.00	8.86 ± 0.00	55.79 ± 0.01	8.04 ± 0.00	76.23 ± 0.00	9.71 ± 0.00	53.53 ± 0.00	17.47 ± 0.00
	3	7.72 ± 0.00	22.87 ± 0.00	5.92 ± 0.00	17.03 ± 0.00	7.60 ± 0.00	18.61 ± 0.00	4.85 ± 0.00	20.17 ± 0.00	6.45 ± 0.00	32.88 ± 0.00	4.24 ± 0.00	63.48 ± 0.02	10.65 ± 0.00	62.30 ± 0.00	12.48 ± 0.00
Inosine	1	110.71 ± 0.00	117.85 ± 0.00	81.51 ± 0.01	181.74 ± 0.00	89.96 ± 0.00	177.02 ± 0.00	62.72 ± 0.00	128.49 ± 0.00	79.13 ± 0.01	115.07 ± 0.00	59.66 ± 0.00	50.35 ± 0.00	28.15 ± 0.00	10.27 ± 0.00	41.06 ± 0.00
	2	128.95 ± 0.00	136.13 ± 0.00	69.73 ± 0.00	159.97 ± 0.00	103.49 ± 0.01	184.19 ± 0.00	72.14 ± 0.00	159.10 ± 0.00	87.23 ± 0.00	65.76 ± 0.00	62.77 ± 0.00	21.41 ± 0.00	22.00 ± 0.00	15.50 ± 0.00	16.20 ± 0.00
	3	141.51 ± 0.00	140.81 ± 0.00	88.91 ± 0.00	193.67 ± 0.01	109.70 ± 0.00	195.66 ± 0.00	61.00 ± 0.00	171.78 ± 0.01	60.51 ± 0.00	80.21 ± 0.00	34.09 ± 0.00	30.13 ± 0.00	32.20 ± 0.00	12.57 ± 0.00	12.54 ± 0.00
IMP	1	102.70 ± 0.00	132.37 ± 0.00	8.43 ± 0.00	8.07 ± 0.00	20.04 ± 0.00	7.43 ± 0.00	4.13 ± 0.00	7.77 ± 0.00	0.00	7.72 ± 0.00	0.00	6.11 ± 0.00	0.00	2.50 ± 0.00	0.00
	2	103.38 ± 0.00	58.67 ± 0.00	19.10 ± 0.00	34.40 ± 0.00	7.13 ± 0.00	5.56 ± 0.00	1.91 ± 0.00	0.00	0.00	0.00	0.00	0.00	0.00	0.00	0.00
	3	120.59 ± 0.01	116.16 ± 0.00	24.71 ± 0.00	47.29 ± 0.00	8.39 ± 0.00	6.28 ± 0.00	0.00	7.03 ± 0.00	0.00	8.37 ± 0.00	0.00	4.66 ± 0.00	0.00	3.00 ± 0.00	0.00
Lactate	1	576.51 ± 0.01	613.29 ± 0.01	219.11 ± 0.01	652.26 ± 0.01	250.87 ± 0.01	643.03 ± 0.01	182.97 ± 0.00	468.45 ± 0.01	225.64 ± 0.03	527.62 ± 0.00	179.59 ± 0.01	304.69 ± 0.02	116.81 ± 0.01	360.98 ± 0.01	146.79 ± 0.00
	2	588.61 ± 0.00	506.47 ± 0.00	180.55 ± 0.00	545.75 ± 0.00	253.77 ± 0.00	568.22 ± 0.02	178.19 ± 0.00	485.83 ± 0.02	221.04 ± 0.01	454.54 ± 0.02	167.49 ± 0.00	365.82 ± 0.01	102.09 ± 0.01	159.40 ± 0.01	143.21 ± 0.01
	3	633.48 ± 0.01	578.85 ± 0.00	207.80 ± 0.00	674.83 ± 0.00	278.22 ± 0.01	593.79 ± 0.03	147.24 ± 0.01	530.04 ± 0.00	153.36 ± 0.01	218.87 ± 0.00	102.29 ± 0.02	163.72 ± 0.01	137.20 ± 0.01	280.87 ± 0.00	48.65 ± 0.00
Mannitol	1	0.00	0.00	303.00 ± 0.01	0.00	142.25 ± 0.01	0.00	147.36 ± 0.01	0.00	223.74 ± 0.01	0.00	642.18 ± 0.04	0.00	112.66 ± 0.01	0.00	144.45 ± 0.03
	2	0.00	0.00	257.48± 0.02	0.00	168.25 ± 0.01	0.00	229.00 ± 0.01	0.00	513.98 ± 0.04	0.00	514.37 ± 0.02	0.00	382.38 ± 0.02	0.00	236.53 ± 0.01
	3	0.00	0.00	125.65 ± 0.00	0.00	219.55 ± 0.01	0.00	366.80 ± 0.01	0.00	459.45 ± 0.02	0.00	172.09 ± 0.01	0.00	530.34 ± 0.01	0.00	257.82 ± 0.01
Nicotinate	1	6.89 ± 0.00	7.14 ± 0.00	2.79 ± 0.00	7.93 ± 0.00	3.26 ± 0.00	8.57 ± 0.00	2.46 ± 0.00	7.16 ± 0.00	3.87 ± 0.00	7.69 ± 0.00	2.52 ± 0.00	7.12 ± 0.00	2.05 ± 0.00	7.14 ± 0.00	2.82 ± 0.00
	2	7.26 ± 0.00	6.32 ± 0.00	2.75 ± 0.00	7.03 ± 0.00	3.87 ± 0.00	8.34 ± 0.00	2.80 ± 0.00	7.46 ± 0.00	3.69 ± 0.00	7.98 ± 0.00	2.87 ± 0.00	7.89 ± 0.00	1.50 ± 0.00	6.82 ± 0.00	2.80 ± 0.00
	3	7.93 ± 0.00	8.02 ± 0.00	3.05 ± 0.00	8.03 ± 0.00	3.55 ± 0.00	7.82 ± 0.00	2.31 ± 0.00	8.02 ± 0.00	1.94 ± 0.00	7.84 ± 0.00	2.45 ± 0.00	6.32 ± 0.00	2.10 ± 0.00	6.94 ± 0.00	1.74 ± 0.00
Phenylalanine	1	3.63 ± 0.00	5.46 ± 0.00	2.34 ± 0.00	4.39 ± 0.00	3.63 ± 0.00	5.81 ± 0.00	3.38 ± 0.00	5.53 ± 0.00	5.38 ± 0.00	6.40 ± 0.00	2.99 ± 0.00	11.67 ± 0.00	4.24 ± 0.00	11.34 ± 0.00	6.02 ± 0.00
	2	2.98 ± 0.00	6.03 ± 0.01	3.01 ± 0.00	5.00 ± 0.00	3.01 ± 0.00	6.59 ± 0.00	3.51 ± 0.00	6.15 ± 0.00	4.53 ± 0.00	8.81 ± 0.00	3.69 ± 0.00	17.10 ± 0.00	2.08 ± 0.00	27.77 ± 0.00	4.42 ± 0.00
	3	4.66 ± 0.00	5.23 ± 0.00	3.29 ± 0.00	5.23 ± 0.00	3.57 ± 0.00	6.45 ± 0.00	2.46 ± 0.00	5.54 ± 0.00	3.82 ± 0.00	7.21 ± 0.00	2.80 ± 0.00	13.36 ± 0.00	3.89 ± 0.00	18.58 ± 0.00	4.92 ± 0.00
Succinate	1	1.37 ± 0.00	0.18 ± 0.00	8.51 ± 0.00	0.00	3.11 ± 0.00	0.00	3.70 ± 0.00	0.17 ± 0.00	2.49 ± 0.00	1.47 ± 0.00	1.86 ± 0.00	4.41 ± 0.00	0.34 ± 0.00	21.26 ± 0.00	0.92 ± 0.00
	2	0.86 ± 0.00	0.22 ± 0.00	4.94 ± 0.00	0.00	3.17 ± 0.00	0.00	4.60 ± 0.00	0.08 ± 0.00	2.90 ± 0.00	5.64 ± 0.00	3.43 ± 0.00	19.07 ± 0.00	1.39 ± 0.00	7.68 ± 0.00	3.82 ± 0.00
	3	0.36 ± 0.00	0.00	5.48 ± 0.00	0.00	4.86 ± 0.00	0.00	4.44 ± 0.00	0.11 ± 0.00	5.46 ± 0.00	0.29 ± 0.00	0.87 ± 0.00	1.98 ± 0.00	4.99 ± 0.00	14.35 ± 0.00	1.29 ± 0.00
TMA	1	0.52 ± 0.00	0.15 ± 0.00	0.22 ± 0.00	0.36 ± 0.00	0.11 ± 0.00	0.95 ± 0.00	1.17 ± 0.00	1.90 ± 0.00	0.23 ± 0.00	14.83 ± 0.00	0.37 ± 0.00	17.18 ± 0.01	0.61 ± 0.00	40.75 ± 0.01	0.72 ± 0.00
	2	0.08 ± 0.00	0.24 ± 0.00	0.06 ± 0.00	0.29 ± 0.00	0.09 ± 0.00	0.28 ± 0.00	0.05 ± 0.00	1.22 ± 0.00	0.10 ± 0.00	26.85 ± 0.01	0.30 ± 0.00	38.96 ± 0.00	0.40 ± 0.00	23.07 ± 0.00	2.39 ± 0.00
	3	0.26 ± 0.00	0.20 ± 0.00	0.13 ± 0.00	0.47 ± 0.00	0.28 ± 0.00	0.90 ± 0.00	0.13 ± 0.00	1.01 ± 0.00	0.12 ± 0.00	2.70 ± 0.00	0.34 ± 0.00	14.82 ± 0.00	0.81 ± 0.00	34.35 ± 0.00	1.72 ± 0.00
TMAO	1	50.85 ± 0.00	51.30 ± 0.02	21.25 ± 0.01	44.16 ± 0.02	18.08 ± 0.00	55.81 ± 0.04	12.02 ± 0.00	54.43 ± 0.01	21.28 ± 0.00	27.32 ± 0.01	14.38 ± 0.01	24.97 ± 0.01	19.72 ± 0.01	0.00	17.87 ± 0.01
	2	53.33 ± 0.02	66.23 ± 0.01	21.56 ± 0.00	59.66 ± 0.01	23.96 ± 0.01	55.07 ± 0.02	15.66 ± 0.01	58.93 ± 0.00	22.83 ± 0.00	17.42 ± 0.01	19.00 ± 0.01	3.04 ± 0.00	14.68 ± 0.01	28.60 ± 0.01	16.46 ± 0.02
	3	65.95 ± 0.02	49.95 ± 0.00	18.93 ± 0.02	55.08 ± 0.00	21.84 ± 0.01	55.27 ± 0.03	13.99 ± 0.01	53.69 ± 0.01	11.97 ± 0.00	53.36 ± 0.02	19.77 ± 0.01	29.86 ± 0.03	15.13 ± 0.01	3.63 ± 0.00	13.61 ± 0.01
Tyrosine	1	6.41 ± 0.00	8.26 ± 0.00	2.65 ± 0.00	6.88 ± 0.00	5.24 ± 0.00	8.01 ± 0.00	3.77 ± 0.00	7.35 ± 0.00	6.04 ± 0.00	8.51 ± 0.00	2.81 ± 0.00	11.75 ± 0.00	4.76 ± 0.00	12.82 ± 0.00	6.32 ± 0.00
	2	6.64 ± 0.00	6.46 ± 0.00	3.95 ± 0.00	6.93 ± 0.00	4.11 ± 0.00	8.87 ± 0.00	4.13 ± 0.00	7.50 ± 0.00	4.69 ± 0.00	10.50 ± 0.00	3.85 ± 0.00	18.82 ± 0.01	2.15 ± 0.00	30.84 ± 0.00	4.30 ± 0.00
	3	7.98 ± 0.00	8.44 ± 0.00	4.80 ± 0.00	10.25 ± 0.00	5.97 ± 0.00	10.00 ± 0.00	3.74 ± 0.00	8.57 ± 0.00	4.12 ± 0.00	10.24 ± 0.00	3.73 ± 0.00	11.19 ± 0.00	4.24 ± 0.00	8.29 ± 0.00	4.92 ± 0.00
Valine	1	8.19 ± 0.00	9.76 ± 0.00	5.07 ± 0.00	9.39 ± 0.00	5.16 ± 0.00	12.16 ± 0.00	4.73 ± 0.00	8.95 ± 0.00	7.84 ± 0.00	9.98 ± 0.00	7.03 ± 0.00	15.58 ± 0.00	6.86 ± 0.00	14.50 ± 0.00	8.59 ± 0.01
	2	9.50 ± 0.00	10.32 ± 0.00	5.34 ± 0.00	9.83 ± 0.00	6.01 ± 0.00	11.87 ± 0.00	5.41 ± 0.00	9.89 ± 0.00	7.21 ± 0.00	13.29 ± 0.00	6.08 ± 0.00	23.15 ± 0.00	4.04 ± 0.00	33.22 ± 0.01	8.51 ± 0.00
	3	11.17 ± 0.01	10.94 ± 0.00	5.62 ± 0.00	12.37 ± 0.00	7.57 ± 0.00	12.11 ± 0.00	5.12 ± 0.00	11.16 ± 0.00	6.31 ± 0.00	12.79 ± 0.00	6.38 ± 0.00	18.18 ± 0.00	7.07 ± 0.00	22.43 ± 0.00	8.71 ± 0.00
**Experiment 2. (Evaluation)**																
**Compound**	**7**		**9**		**11**			**14**		**17**		**19**		**21**		
	**RS-2**		**RS-2**	**STS-2**	**RS-2**		**STS-2**	**RS-2**	**STS-2**	**RS-2**	**STS-2**	**RS-2**	**STS-2**	**RS-2**		**STS-2**
2,3-butanediol	0.12 ± 0.00		1.27 ± 0.00	4.72 ± 0.00	96.78 ± 0.00		57.37 ± 0.00	119.28 ± 0.01	111.50 ± 0.01	131.18 ± 0.01	95.74 ± 0.00	124.69 ± 0.01	74.89 ± 0.02	124.23 ± 0.00		90.04 ± 0.01
Acetoin	0.00		4.47 ± 0.00	7.78 ± 0.00	24.47 ± 0.00		25.18 ± 0.00	17.39 ± 0.00	37.21 ± 0.01	27.14 ± 0.01	45.75 ± 0.02	25.78 ± 0.00	33.76 ± 0.00	22.95 ± 0.00		44.56 ± 0.01
Alanine	65.42 ± 0.00		71.11 ± 0.01	76.36 ± 0.00	88.59 ± 0.00		76.97 ± 0.01	86.78 ± 0.00	84.91 ± 0.00	82.55 ± 0.01	83.95 ± 0.00	85.54 ± 0.00	87.61 ± 0.01	87.10 ± 0.01		84.53 ± 0.01
Anserine	580.57 ± 0.02		488.55 ± 0.01	556.42 ± 0.01	603.14 ± 0.00		477.29 ± 0.00	679.32 ± 0.01	540.87 ± 0.01	596.26 ± 0.01	433.81 ± 0.01	618.26 ± 0.00	423.81 ± 0.00	646.31 ± 0.01		378.07 ± 0.00
β-Alanine	19.78 ± 0.00		18.61 ± 0.00	19.46 ± 0.00	24.97 ± 0.00		18.80 ± 0.00	27.23 ± 0.00	24.24 ± 0.00	24.81 ± 0.00	22.29 ± 0.00	24.12 ± 0.01	22.62 ± 0.00	25.81 ± 0.01		25.63 ± 0.00
Betaine	12.04 ± 0.01		14.61 ± 0.00	11.34 ± 0.00	13.48 ± 0.00		8.47 ± 0.00	13.30 ± 0.00	9.96 ± 0.00	13.53 ± 0.00	8.21 ± 0.00	11.54 ± 0.00	7.50 ± 0.00	12.72 ± 0.00		10.76 ± 0.00
Creatine (mmol/100 g)	3.93 ± 0.02		3.64 ± 0.00	3.27 ± 0.04	4.06 ± 0.03		2.80 ± 0.01	4.27 ± 0.02	3.49 ± 0.01	3.91 ± 0.03	2.90 ± 0.01	3.96 ± 0.03	2.80 ± 0.02	3.99 ± 0.02		2.89 ± 0.03
Ethanol	2.64 ± 0.00		1.75 ± 0.00	2.03 ± 0.00	15.73 ± 0.01		3.83 ± 0.01	20.18 ± 0.00	5.20 ± 0.00	15.46 ± 0.00	6.99 ±0.01	20.36 ± 0.00	9.59 ± 0.00	21.87 ± 0.00		8.75 ± 0.00
Formate	0.26 ± 0.00		0.65 ± 0.00	1.50 ± 0.00	12.87 ± 0.00		23.55 ± 0.00	0.82 ± 0.00	28.67 ± 0.00	0.81 ± 0.00	19.47 ± 0.00	0.50 ± 0.00	23.84 ± 0.00	0.95 ± 0.00		4.07 ± 0.00
Fumarate	0.43 ± 0.00		*	*	0.34 ± 0.00		*	0.47 ± 0.00	*	0.49 ± 0.00	*	0.50 ± 0.00	*	*		*
Glutamate	15.01 ± 0.00		17.03 ± 0.00	17.02 ± 0.00	18.99 ± 0.00		17.15 ± 0.00	10.47 ± 0.00	14.58 ± 0.00	7.00 ± 0.00	13.65 ± 0.00	6.84 ± 0.00	7.75 ± 0.00	6.15 ± 0.00		10.77 ± 0.00
Glycine	47.41 ± 0.00		45.61 ± 0.01	38.73 ± 0.01	52.61 ± 0.02		33.37 ± 0.01	50.40 ± 0.01	40.72 ± 0.01	52.27 ± 0.02	36.42 ± 0.00	54.39 ± 0.01	34.79 ± 0.02	55.81 ±0.03		44.09 ± 0.02
Histamine	0.00		0.00	0.00	31.99 ± 0.01		22.07 ± 0.01	41.32 ± 0.00	33.61 ± 0.00	32.18 ± 0.00	27.47 ± 0.00	32.41 ± 0.01	29.47 ± 0.01	34.10 ± 0.00		30.07 ± 0.00
Hypoxanthine	17.23 ± 0.00		26.54 ± 0.00	28.76 ± 0.00	88.21 ± 0.00		45.95 ± 0.00	93.83 ± 0.01	42.26 ± 0.00	80.06 ± 0.00	21.36 ± 0.00	82.82 ± 0.00	26.51 ± 0.00	85.22 ± 0.00		28.13 ± 0.01
Inosine	116.04 ± 0.00		121.68 ± 0.00	116.28 ± 0.00	8.05 ± 0.00		6.05 ± 0.00	0.00	0.00	0.00	0.00	0.00	0.00	0.00		0.00
IMP	70.35 ± 0.00		1.88 ± 0.00	0.00	0.00		0.00	0.00	0.00	0.00	0.00	0.00	0.00	0.00		0.00
Mannitol	0.00		0.00	52.41 ± 0.00	0.00		151.47 ± 0.01	0.00	114.94 ± 0.01	0.00	139.94 ± 0.01	0.00	185.30 ± 0.00	0.00		94.09 ± 0.00
Nicotinate	5.69 ± 0.00		4.99 ± 0.00	5.80 ± 0.00	6.98 ± 0.00		5.17 ± 0.00	8.18 ± 0.00	6.68 ± 0.00	7.03 ± 0.00	5.37 ± 0.00	6.91 ± 0.00	5.17 ± 0.00	7.82 ± 0.00		5.37 ± 0.00
Putrescine	0.00		0.00	0.00	0.00		0.00	4.74 ± 0.00	3.38 ± 0.00	7.22 ± 0.00	3.24 ± 0.01	8.43 ± 0.00	6.60 ± 0.00	9.04 ± 0.00		5.53 ± 0.00
Succinate	0.21 ± 0.00		0.13 ± 0.00	0.61 ± 0.00	3.10 ± 0.00		4.09 ± 0.00	9.46 ± 0.01	9.68 ± 0.00	10.09 ± 0.01	17.19 ± 0.01	14.81 ± 0.01	20.24 ± 0.00	22.49 ± 0.00		21.96 ± 0.00
TMA	0.28 ± 0.00		6.91 ± 0.00	8.57 ± 0.00	22.38 ± 0.02		20.14 ± 0.01	32.75 ± 0.01	22.69 ± 0.01	35.84 ± 0.02	24.96 ± 0.01	39.85 ± 0.01	24.25 ± 0.00	46.36 ± 0.01		27.69 ± 0.00
TMAO	17.43 ± 0.01		14.08 ± 0.01	9.07 ± 0.01	0.00		1.49 ± 0.00	0.00	0.00	0.46 ± 0.01	0.21 ± 0.01	0.08 ± 0.00	0.63 ± 0.01	0.00		0.40 ± 0.00
Tyramine	0.00		0.00	0.00	2.34 ± 0.00		2.27 ± 0.00	3.00 ± 0.00	3.39 ± 0.00	2.68 ± 0.00	3.61 ± 0.00	3.72 ± 0.00	5.00 ± 0.00	4.36 ± 0.00		3.51 ± 0.00
Tyrosine	7.00 ± 0.00		5.82 ± 0.00	7.60 ± 0.00	0.95 ± 0.00		1.36 ± 0.00	0.75 ± 0.00	0.84 ± 0.00	1.32 ± 0.00	1.11 ± 0.00	1.01 ± 0.00	1.14 ± 0.00	1.07 ± 0.00		1.03 ± 0.00
Uracil	0.67 ± 0.00		0.58 ± 0.00	0.70 ± 0.00	0.00		0.00	0.00	0.53 ± 0.00	0.36 ± 0.00	1.14 ± 0.00	0.82 ± 0.00	1.45 ± 0.00	1.13 ± 0.00		1.18 ± 0.00

## Abbreviations

ADP	Adenosine diphosphate
Ala	Alanine
AMP	Adenosine monophosphate
Asp	Asparate/aspartic acid
ATP	Adenosine triphosphate
b-Ala	β-Alanine
Cr/PCr	Creatine/phosphocreatine
D_2_O	Deuterium oxide
Glu	Glutamate/glutamic acid
Gly	Glycine
Glyco	Glycogen
GPC	Glycerophosphocholine
Hx	Hypoxanthine
Hz	Hertz
Ile	Isoleucine
IMP	Inosine-5′-monophosphate
Ino	Inosine
LA	Lactate/lactic acid
Leu	Leucine
Mal	Maltose
Man	Mannitol
Met	Methionine
NA	Nicotinate/nicotinic acid
NAM	Niacinamide
NMR	Nuclear Magnetic Resonance
NS	Number of scans
PCA	Principal component analysis
Phe	Phenylalanine
ppm	Parts per million
RG	Receiver gain
RS	Reference samples
*S. latissima*	*Saccharina latissima*
STS	Seaweed-treated samples
SW	Spectral width
Tau	Taurine
TCA	Trichloroacetic acid
Thr	Threonine
TMA	Trimethylamine
TMAO	Trimethylamine oxide
TMS	Tetramethylsilane
TSP	Trimethylsilylpropionic acid
Tyr	Tyrosine
Ura	Uracil
Val	Valine
